# Mitochondrial Transplantation as a Novel Therapeutic Strategy for Mitochondrial Diseases

**DOI:** 10.3390/ijms22094793

**Published:** 2021-04-30

**Authors:** Anna Park, Mihee Oh, Su Jeong Lee, Kyoung-Jin Oh, Eun-Woo Lee, Sang Chul Lee, Kwang-Hee Bae, Baek Soo Han, Won Kon Kim

**Affiliations:** 1 Metabolic Regulation Research Center, Korea Research Institute of Bioscience and Biotechnology (KRIBB), Daejeon 34141, Korea; annapark@kribb.re.kr (A.P.); krystallee@kribb.re.kr (S.J.L.); kjoh80@kribb.re.kr (K.-J.O.); ewlee@kribb.re.kr (E.-W.L.); lesach@kribb.re.kr (S.C.L.); khbae@kribb.re.kr (K.-H.B.); 2 Biodefense Research Center, Korea Research Institute of Bioscience and Biotechnology (KRIBB), Daejeon 34141, Korea; ohmihee@kribb.re.kr

**Keywords:** mitochondria, mitochondrial function, mitochondrial dysfunction, mitochondrial disease, mitochondrial transplantation

## Abstract

Mitochondria are the major source of intercellular bioenergy in the form of ATP. They are necessary for cell survival and play many essential roles such as maintaining calcium homeostasis, body temperature, regulation of metabolism and apoptosis. Mitochondrial dysfunction has been observed in variety of diseases such as cardiovascular disease, aging, type 2 diabetes, cancer and degenerative brain disease. In other words, the interpretation and regulation of mitochondrial signals has the potential to be applied as a treatment for various diseases caused by mitochondrial disorders. In recent years, mitochondrial transplantation has increasingly been a topic of interest as an innovative strategy for the treatment of mitochondrial diseases by augmentation and replacement of mitochondria. In this review, we focus on diseases that are associated with mitochondrial dysfunction and highlight studies related to the rescue of tissue-specific mitochondrial disorders. We firmly believe that mitochondrial transplantation is an optimistic therapeutic approach in finding a potentially valuable treatment for a variety of mitochondrial diseases.

## 1. Mitochondrial Function

### 1.1. General Roles and Dynamics of Mitochondria

Mitochondria manage diverse aspects of cellular function by providing the required supply of adenosine triphosphate (ATP), regulating Ca^2+^ signaling and controlling reactive oxygen species (ROS) levels [1,2]. In particular, they are mainly present in muscle cells and major body organs such as the liver, heart and brain that require energy [3]. Mitochondria are also flexible as they can switch from regulating normal cell functions to promoting apoptosis when required. They play a central role in necrosis and apoptosis [4].

Mitochondria have a life cycle in which their dynamics and mitophagy contribute to quality control. Depending on the signals they receive, they undergo fusion or fission. These fusion and fission events are collectively referred to as mitochondrial dynamics. Many signaling proteins are involved in these processes. Mitofusins 1 and 2 (MFN1 and MFN2) are known to be involved in the fusion of the mitochondrial outer membrane, and optic atrophy type 1 (Opa1) is known to induce the fusion of the mitochondrial inner membrane. As for fission of the outer mitochondrial membrane, it is regulated by dynamin-related protein 1 (Drp1) and fission 1 (Fis1) [5,6].

### 1.2. Tissue Specificity of Mitochondrial Function

Interestingly, just like differentiated cells, mitochondria carry out specialized functions unique to specific tissues. For example, mitochondria in the liver are primarily involved in biosynthetic functions, and mitochondria in the heart or muscle mainly perform ATP production. In addition, mitochondria in adipocytes are crucial in regulating adipocyte differentiation, insulin sensitivity and adaptive thermogenesis [7]. An analysis of the mitochondrial proteome isolated from various tissues such as the brain, liver, heart and kidney of rats showed mitochondrial heterogeneity, specializing in different functions between tissues. The authors of this study speculated that tissue-specific mitochondrial function is regulated by tissue-specific nuclear-encoded proteins [8]. Another possible way to modulate the specificity of mitochondria is the control of intercellular Ca^2+^ concentration. In addition to energy production, mitochondria are involved in various signaling pathways in regulating Ca^2+^ flux in living cells. It has been suggested previously that there are different ways in which mitochondria control the flow of Ca^2+^ depending on the type of tissues. This evidence indicates that there are tissue-specific roles of mitochondria that need to be studied further [9].

**Figure 1:**
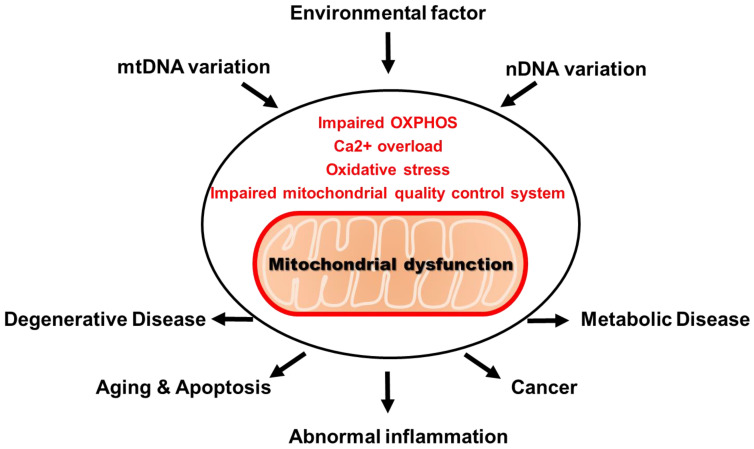
Mitochondrial dysfunction and disease.

Abnormality in mitochondria disrupts major physiological functions, such as ATP production, oxidative phosphorylation, ROS production and Ca^2+^ regulation. These are considered to be mitochondrial dysfunction [10]. More than 100 mitochondrial diseases caused by mitochondrial defects have been identified, including obesity, diabetes, heart disease, neurodegenerative disease and aging. A recent study has shown that there is a cell-specific response induced by defects in mitochondrial proteins or mitochondrial DNA (mtDNA) [11]. As a result of this, the development of various types of mitochondrial diseases depend on tissue-specific mitochondrial dysfunction (Figure 1) [12,13,14,15].

## 2. Mitochondrial Dysfunction and Disease

### 2.1. Mitochondrial Dysfunction in Neurological Disease

Mitochondrial abnormality causes a variety of neurological diseases, such as ischemic brain disease, Parkinson’s disease, Alzheimer’s disease, stroke and mitochondrial encephalopathy [16,17,18,19,20]. However, their etiology and the role of mitochondria in the pathogenesis have not yet been clearly elucidated.

Alzheimer’s disease (AD) is a brain disease with a pathological feature and progressive impairment of memory and cognitive abilities. AD is distinguished by the presence of extracellular neurogenic plaques and intracellular nerve fiber tangles. Amyloid β peptide (Aβ) is the main component of plaques, and the entanglement is made up of hyperphosphorylated tau protein. Several studies have shown abnormal expression of Drp1 in the postmortem brains of patients with AD, AD mouse models and AD in vitro models. An elevated level of Drp1 in AD and other neurodegenerative diseases is known to cause excessive mitochondria fission, which leads to mitochondrial dysfunction and neuronal damage [21,22]. Hence, these results imply that abnormal mitochondrial dynamics may be the cause of AD.

Parkinson’s disease (PD) is the second most common neurodegenerative disorder. Pathologically, it is characterized by the loss of dopaminergic neurons in the black matter and the presence of Lewy bodies, which are abnormal protein aggregates consisting of the presynaptic protein α-synuclein [23]. Damaged mitochondria are selectively removed by mitophagy. Mitochondrial quality control is crucial in maintaining the survival of neurons. Activation of parkin and PTEN-induced kinase 1 (PINK1) plays a direct role in the mitochondrial quality control pathway of cells [17,18]. When these genes are mutated, it causes abnormal quality control of mitochondria, which leads to neurodegeneration [24,25]. Additionally, it was also found that actual PD patients lacked mitochondrial NADH dehydrogenase (complex I) activity [26]. Consistent with this result, inhibition of OXPHOS complex I by treatment with chemicals such as rotenone and 1-methyl-4-phenyl-1,2,3,6-tetrahydropyridine (MPTP) results in neuropathological and behavioral symptoms similar to human PD [27].

Huntington’s disease (HD) is a progressive neurodegenerative disorder that is inherited primarily due to an abnormal CAG repeat expansion of the huntingtin (HTT) gene. In pathology of HD involves a gradual loss of intermediate spiny neurons in the striatum, cortical atrophy and degeneration of other brain regions. In the brain tissues of HD patients and HTT-knockout mice, it was observed that the number of mitochondria was decreased and mitochondrial respiration and ATP production were significantly impaired [28]. In addition, abnormal mitochondrial dynamics such as an increase in Drp1 and a decrease in Mfn1 were observed [29].

Amyotrophic lateral sclerosis (ALS) is a devastating neurodegenerative disease affecting motor neurons, resulting in muscle weakness and atrophy and ultimately death. Several studies reported that mitochondrial dysfunction in ALS animal models is selectively associated with superoxide dismutase 1 (SOD1) mutation. Misfolded SOD1 protein by SOD1 mutation was found in areas affected by ALS and is considered to mediate toxicity. However, the explicit link between ALS and SOD1 has not been revealed [30,31]. In mice with **ischemic stroke**, mitochondrial fission was shown to be an early pathological feature and this was accompanied by morphological changes in mitochondria, production of high levels of free radicals and ATP depletion [32].

### 2.2. Mitochondrial Dysfunction in Type 2 Diabetes

Tissues such as the liver or skeletal muscle that use carbohydrates as an energy source absorb glucose from the blood by stimulation of insulin secreted from pancreatic β cells. Diabetes is caused by the absence or insufficient production of insulin, or an inability to use insulin [33]. This results in a high glucose level in the blood.

The molecular mechanisms involved in insulin-stimulated glucose transporter type 4 (GLUT4) translocation and glucose transport are well known. In brief, insulin receptor (IR) binding of insulin induces conformational changes of the receptor, resulting in auto-phosphorylation and activation of receptor tyrosine kinases, which in turn recruit and stimulate insulin receptor substrate-1 (IRS-1). Activated IRS-1 continuously activates serine/threonine kinase 2 (AKT2) through interaction with phosphoinositide 3-kinase (PI3K), which ultimately induces translocation of GLUT4 from the cytoplasm to the plasma membrane, thereby increasing glucose uptake [34]. In this process, fatty acids act as a prominent inhibitor of glucose absorption [35]. An increase in plasma fatty acids increases intracellular fatty acyl-CoA and diacylglycerol (DAG) concentration in skeletal muscle. This induces the activation of protein kinase C-δ (PKC-δ), which reduces the activation of IRS-1-associated PI3K through the phosphorylation of IRS-1 at Ser^307^. This leads to a decrease in insulin-stimulated glucose transport activity [36].

Based on this, it is accepted that mitochondrial defects play an important role in the development of insulin resistance. Accumulation of spontaneous mtDNA mutations caused by aging or environmental factors induces mitochondrial β-oxidation defects [37]. These defects in β-oxidation increase fatty acids levels, and induce insulin resistance through inhibition of translocation of GLUT4 [38]. Skeletal muscle cells of type 2 diabetes patients had a smaller size and fewer mitochondria than those of normal people, and showed reduced electron transport chain (ETC) activity [39]. In addition, mtDNA mutation can induce inhibition of mitochondrial ATP-generating capacity, which in turn leads to a deficiency of ATP required for glucose transport, which can increase insulin resistance [40].

### 2.3. Mitochondrial Dysfunction in Hepatic Disease

The liver carries out a variety of crucial biological functions that regulate the homeostasis of glucose, fatty acids and amino acids and the synthesis of plasma proteins such as albumin. It also acts to detoxify harmful metabolites that cells are continuously exposed to. Most of these liver functions are dependent on the energy produced by mitochondria. Thus, mitochondrial dysfunction in liver tissue triggers various hepatic diseases such as fatty liver disease, hepatitis and liver cancer [41].

Non-alcoholic fatty liver disease (NAFLD) is one of the most common chronic liver diseases, characterized by the accumulation of lipids in hepatocytes, that is not linked with excessive alcohol intake. Mitochondrial dysregulation due to ETC defects are considered to be a decisive etiology of NAFLD. Decreased fatty acid oxidation due to mitochondrial defects favors lipid accumulation and induces overproduction of ROS that contribute to necrotic inflammation [42,43].

Recently, it has been reported that mitochondrial uncoupling plays a crucial role in the pathogenesis of liver disease by reducing mitochondrial proton motive force and ATP production [44]. Therefore, mitotherapy can be an effective strategy for treating liver disorders.

## 3. Mitochondrial Transplantation for Therapeutic Use

### 3.1. Mitochondrial Replacement Therapy (MRT)

In recent years, advances in molecular and biochemical methodologies have led to a better understanding of mitochondrial defects and their mechanisms as the cause of various diseases, but therapies for mitochondrial disorders are still insufficient. Several drugs have been evaluated to improve mitochondrial function and the symptoms of mitochondrial dysfunction. Agents such as coenzyme Q10, idebenone, riboflavin, dichloroacetate and thiamine were used to improve function of the ETC, and creatine monohydrate was used as an energy shuttle for high-energy phosphate movement from mitochondria to the cytoplasm. In addition, antioxidants such as vitamin C, vitamin E and lipoic acid have been tested as supplements to elucidate mitochondrial disorders, and a number of other drugs are the subjects of ongoing clinical trials [45,46,47,48,49,50,51,52]. However, these agents can provide very limited protection, and most mitochondrial diseases are considered irreversible as they are caused by injury, such as mtDNA mutation [52]. Due to this, therapies involving these agents have limitations.

Mitochondrial transplantation is an innovative strategy for the treatment of mitochondrial dysfunction to overcome the limitations of therapies using agents. Mitochondrial transplantation aims to transfer functional exogenous mitochondria into mitochondrion-defective cells for recovery or prevention of mitochondrial diseases. Simply put, replacing an old engine with a new one to regain its function.

Mitochondrial transfer was first attempted by Clark and Shay. They co-incubated mitochondria purified from cells resistant to chloramphenicol and efrapeptin and mammalian cells sensitive to these antibiotics. As a result, mitochondrion-mediated transfer of antibiotic resistance via endocytosis was confirmed [53]. This phenomenon of delivering organelles to recipient cells can be applied to mitochondrial diseases. Replacement of non-functional mitochondria in damaged tissues or cells with functional ones could possibly be a new approach.

Several studies have shown in vitro that the intercellular transfer of mitochondria occurs naturally. When DsRed-labeled mitochondria isolated from human uterine endometrial gland-derived mesenchymal cells (EMCs) were co-incubated with isogenic EMCs for 24 h, an accumulation of exogenous mitochondria in the cytoplasm of recipients was observed through live fluorescence cell imaging [54]. In another study, it was also observed that a xenogenic transfer of mitochondria isolated from mouse liver tissue into human cells lacking functional mitochondria (ρ^0^ cells) restored respiration function [55]. These results prove the possibility of treating mitochondrial diseases through mitochondrial transplantation.

### 3.2. Mitochondrial Transplantation in Neurological Diseases

Recently, a considerable number of studies demonstrated the effectiveness of mitochondrial transplantation in various diseases. There are many reports of mitotherapy in tissues, animal models and even in patients, as well as in vitro. These include neurological diseases, drug-induced liver toxicity and liver disease, including fatty liver and myocardial ischemia–reperfusion injury. Here, we describe studies of mitochondrial transplantation for the treatment of various mitochondrial disorders. Table 1 and Table 2 summarize the mitochondrial source, target cell/organ, therapeutic outcome, delivery method and mitochondrial concentration range in various mitochondrial disease models (Table 1 and Table 2).

Several studies have evaluated the improvement in mitochondrial function via mitochondrial transfer in neurological disease models. Ailing Fu and colleagues found that mitochondria isolated from human hepatoma cells (HepG2 cells) could naturally enter human neuroblastoma cells (SH-SY5Y cells). The transferred mitochondria increased ATP content and reduced ROS production and apoptosis in an in vitro PD model [56]. Furthermore, they intravenously injected mitochondria isolated from human hepatoma cells (HepG2 cells) into neurotoxin-induced PD mouse brain. The recipient mouse suppressed PD progression by increasing the activity of the ETC, and reduced free radical generation and apoptotic cells [56].

To increase mitochondrial delivery efficiency, more advanced techniques have been used. One study showed the enhanced delivery and functionality of allogenic exogenous mitochondria using peptide-mediated delivery by conjugating a cell penetrating peptide, Pep-1. When they co-incubated mitochondria with Pep-1 in PD model rat PC12 cells, the internalized mitochondria removed the neurotoxin-induced oxidative stress and apoptotic cell death [57]. The result of transplanting Pep-1-labeled mitochondria into brain tissues of a PD rat model demonstrated that mitochondrial complex I protein and mitochondrial dynamics were restored in dopaminergic neurons, which also improved oxidative DNA damage. The removal of dopaminergic neuron degeneration due to a neurotoxin was also observed in the PD rat model [57].

Another group showed that synaptosomes can be a natural vehicle for the delivery of mitochondria into the cytoplasm of neuronal cells. The transferred healthy mitochondria by synaptosomes restored mitochondrial function in human neuroblastoma cells (LAN5 cells) containing rotenone or carbonyl cyanide m-chlorophenyl hydrazone (CCCP)-damaged mitochondria [58].

### 3.3. Mitochondrial Transplantation in Diabetes, Hepatic and Heart Disease

Mitochondrial transfer also demonstrated a therapeutic effect on diabetic neuropathy. Transfer of mitochondria obtained from bone marrow-derived mesenchymal stem cells (BM-MSCs) to renal proximal tubular epithelial cells (PTECs) of a diabetic neuropathy-induced animal model led to a decrease in ROS production and apoptotic cells [59].

Ailing Fu and co-workers transplanted mitochondria isolated from human hepatoma cells (HepG2 cells) into primary hepatocytes of mouse with acetaminophen (APAP)-induced liver injury. It is well known that drug-induced liver injury causes mitochondrial dysfunction [60,61]. This mitochondrial treatment increased energy supply, reduced ROS production and consequently rescued liver function from APAP-induced hepatotoxicity. This study suggested that exogenous mitochondria could be an effective therapeutic strategy in treating drug-induced liver injury [62].

Mitochondria transplantation was also proved to improve liver with ischemia–reperfusion injury in rats. Mitochondria were isolated from the left ventricle of rabbits and were delivered into ischemia–reperfusion-injured rat liver via splenic injection. This resulted in amelioration of liver by a decrease in both ROS production level and apoptotic cells [63].

Mitochondrial dysfunction is also a major cause of NAFLD. Ailing Fu and his colleagues injected the mitochondria from human hepatocytes (HepG2 cells) into the liver of mice with fatty liver. This mitotherapy resulted in improvement of energy production and reduction of lipid accumulation and oxidation injury and even restored hepatocyte function in fatty liver of mice. This is a promising result that will initiate a wider therapeutic approach towards NAFLD [64].

The therapeutic effect of mitochondrial transplantation has most often been evaluated in a heart disease model. The McCully group demonstrated that ischemia induces mitochondrial dysfunction, and inhibits cellular viability and recovery of cardiomyocyte functions after reperfusion [65]. They isolated mitochondria from tissue unaffected by ischemia and then injected them into the ischemic zone just before reperfusion. This led to a significant enhancement in post-ischemic functional recovery and cellular viability [66]. Continuing with this discovery, they compared the localization of mitochondria via two different pathways of delivery. (1) Direct injection of human cardiac fibroblast mitochondria into rabbit ischemic heart tissue. (2) Vascular delivery of mitochondria through coronary arteries at the onset of reperfusion. As expected, directly injected mitochondria were localized near the site of delivery, whereas vascularly delivered mitochondria were found to be extensively dispersed throughout the heart, and both provided cardioprotection from ischemia–reperfusion injury [67].

The therapeutic effects of mitochondrial transplantation were confirmed in several animal models and it was also clinically applied in humans. The McCully group performed autologous transplantation of mitochondria isolated from non-ischemic rectus abdominis muscles with injured myocardium to patients with ischemia–reperfusion injury. As a result, four of the five patients showed recovery of ventricular function and did not suffer from short-term complications such as arrhythmia, intra-myocardial hematoma or scarring related to mitochondrial transplantation [68].

**Table 2:** Research reports of mitochondrial transplantation in diabetes and hepatic and heart disease.

Mitochondrial Source	Targeted Cell/Organs	Therapeutic Outcome	Delivery Method	Mitochondrial Concentration Range	Reference
MSCs of rats	Renal PTECs (diabetic neurophathy)	Reduction of ROS production and apoptotic cells	Co-culture	Isolated mitochondria obtained from 1 × 10^6^ MSCs	[59]
Human hepatoma cells (HepG2 cells)	Mouse hepatocytes of APAP-induced liver injury	Rescue of liver function from APAP-induced hepatotoxicity	2 h co-incubation	10 µg/mL	[62]
Left ventricular of rabbits	Rat liver with ischemia–reperfusion injury	Decrease in ROS production and apoptotic cells	Splenic injection	7.7 × 10^5^ ± 1.5 × 10^5^/100 μL	[63]
Human hepatoma cells (HepG2 cells)	High-fat diet-induced mouse fatty liver	Rescue of hepatocyte mitochondrial function	Intravenous injection	0.5 mg/kg	[64]
Left ventricular of rabbits	Rabbit heart with ischemia–reperfusion injury	Enhanced myocardial function following ischemia and enhanced cell viability	Direct injection	7.7 × 10^6^ ± 1.5 × 10^6^/mL (eight 0.1 mL injections)	[66]
Human adult cardiac fibroblasts	Rabbit hearts with ischemia–reperfusion injury	Cardioprotection from ischemia–reperfusion injury.	Direct injection/vascular perfusion	1 × 10^8^/0.8 mL (eight 0.1 mL injections)	[67]
Non-ischemic rectus abdominis muscles (autologous)	RI zone of human heart	Four out of five patients successfully separated from ECMO support	Direct injection	1 × 10^7^ ± 1 × 10^4^ /100 μL	[68]

Mesenchymal stem cells (MSCs); proximal tubular epithelial cells (PTECs); reactive oxygen species (ROS); acetaminophen (APAP); regional ischemia (RI); extracorporeal membrane oxygenation (ECMO).

### 3.4. Immune Response of Mitochondrial Transplantation

Providing functional mitochondria to cells with defects has proved to be a promising strategy but there have been studies questioning the activity of the immune response influenced by transplantation of mitochondria.

In the case of acquired mitochondrial diseases, transplantation of autologous mitochondria does not seem to be a problem as they are derived from the patients’ own cells. The McCully group investigated the immune response and autoimmune response caused by autologous mitochondrial transplantation in a rabbit model of ischemic cardiomyopathy. They first isolated mitochondria from a subject’s pectoralis major muscles and then directly injected these into the regional ischemia (RI) zone of the heart. As expected, there was no significant increase in sensitive markers of inflammation, including TNFa, IL-6 and high-sensitivity C-reactive protein (hsCRP). No anti-mitochondrial antibody was detected either [69]. Consistent with this result, transplantation of autologous mitochondria in a porcine model of ischemia/reperfusion showed no significant response in immunity, such as inflammation and cytokine activation markers [70]. These studies hence conclude that mitochondria derived from patients’ own cells do not induce inflammatory and autoimmune responses in in vivo models.

On the other hand, in the case of congenital mitochondrial diseases, autologous mitochondrial transplantation may not be suitable, because there is some probability that mitochondria in other tissues could be dysfunctional. To tackle this issue, transplantation of functional allogenic mitochondria must be considered. Therefore, research and rigorous discussions regarding immune and autoimmune responses resulting from transplantation of allogenic mitochondria is crucial.

The McCully group showed that autologous mitochondrial transplantation did not induce any immune response in various animal models. They further investigated immune response related to allogenic mitochondrial transplantation. They injected mitochondria isolated from gastrocnemius muscle and quadriceps femoris muscle into syngeneic or allogeneic mice through single and serial intraperitoneal injection. As a result, there was no direct or indirect, acute or chronic alloreactivity, immunological reactivity of certain classes of T lymphocytes against transplanted mitochondria or damage-associated molecular pattern molecule (DAMP) reactions to single or serial injections of either autogenetic or allogeneic mitochondria [71].

In contrast, a study conducted by the Brennan group showed that mitochondrial transplantation by single injection induces immune response. They observed significant early rejection of cardiac allografts. They suggest that extracellular mitochondria activate vascular endothelial cells to increase inflammatory cytokines and chemokines, and the activated vascular endothelial cells accelerate graft rejection by increased T cell adhesion and infiltration into allograft tissues [72,73].

In order to expand the potential and stability of mitochondrial transplantation therapy, further studies and discussions on the outcomes and mechanisms related to the immune response that follows transplantation are required.

### 3.5. Mechanism of Mitochondrial Internalization

Several studies conducted in vivo and in vitro clearly showed that mitochondrial transfer can restore lost mitochondrial function in recipient cells. However, there is still a lack of understanding of the mechanism of mitochondrial transfer into cells or tissues. Several potential mechanisms have been suggested to be involved in mitochondrial internalization [74].

In a culture system, co-incubation was used as a means for mitochondrial transfer. Mitochondrial internalization was similarly time-dependently increased in all cell types through simple co-incubation [75]. In most cell types, mitochondrial internalization was clearly confirmed after 1 h of co-incubation, and increased significantly after 4 and 24 h [55,69,76]. In addition, the increase in mitochondrial uptake in recipient cells led to the improvement of the mitochondrial function of target cells, including increased ATP and increased mitochondrial oxygen consumption [55,76].

It has also been revealed that transplanting DsRed1-labeled exogenous mitochondria into GFP-expressing recipient cells through various methods are possible. These included live fluorescence imaging, and it is suggested that mitochondrial internalization involves macropinocytosis [77,78]. The exogenous mitochondria interact directly with cells, by engulfing mitochondria with cellular extensions, which implies the involvement of macropinocytosis or macropinocytosis-like mechanisms in mitochondrial internalization.

The McCully group suggested that internalization of mitochondria in cardiomyocytes occurs through actin-dependent endocytosis by using specific blockers based on their wide use and established selectively [76,79]. One of the blockers, cytochalasin D, inhibits actin polymerization. They confirmed that the internalization of mitochondria into cardiomyocytes decreased significantly when the mitochondria were pre-incubated with cytochalasin D [76].

Tunneling nanotubes (TNTs) are long, non-adherent actin-based cytoplasmic extensions that mediate intercellular communication, ranging from electrical signaling to transfer of organelles [80,81]. A recent study showed spontaneous mitochondrial transfer via cell-to-cell contact-mediated TNTs from UV-untreated cells to UV-treated cells for ameliorating apoptosis [82]. Lian and colleagues showed an interesting result that the efficiency of mitochondrial delivery differs depending on the source of the mitochondria. They transferred mitochondria from human induced pluripotent stem cell (iPSC)-MSCs and BM-MSCs to mouse heart with anthracycline-induced cardiomyopathy through intramyocardial injection. As a result, the mitochondria from iPSC-MSCs showed superior efficiency of mitochondrial transfer than mitochondria from BM-MSCs. The researchers determined that a high expression of intrinsic mitochondrial Rho GTPase 1 (MIRO1) and a high level of tumor necrosis factor α-induced protein 2 (TNFαIP2) expression in iPSC-MSCs induce better responses to TNFα-induced TNT formation, leading to mitochondrial transfer into cardiomyocytes [83].

To deliver exogenous mitochondria into cells or tissues, various transfer or transplantation techniques including direct microinjection, cell-mediated transfer utilizing TNTs, vesicle-mediated delivery and systemic delivery conjugating mitochondria to a carrier or cell penetrating peptide have been used. However, these methods also have disadvantages and should be optimized according to the type of cell or tissue, so it is still necessary to develop a more attractive delivery method [84]. We expect more advanced and efficient cell- or tissue-specific delivery methods to emerge in the next few years. Therefore, for more major advancements in the field of mitochondrial transplantation, it is required to elucidate the precise mitochondrial incorporation mechanisms and unveil the intricacies of functional incorporation within cells.

## 4. Conclusions

A therapeutic effect by mitochondrion transplantation has been proved to be a potential method in improving mitochondrion-related diseases, as discussed in this review. However, there are several issues that must be overcome in order for disease treatment through mitochondrial transplantation to be effectively applied to humans.

Most studies emphasize that mitochondrial isolation should be completed within a short time at a low temperature, as they are very sensitive and their activity and survivability decrease rapidly. In addition, there is currently no method available for long-term storage of mitochondria, hence, they have to be used immediately upon isolation. Therefore, a protocol of an optimal mitochondrion isolation and storage method that maintains mitochondrial integrity and provides longer survivability must be established to ensure feasibility for clinical use. As this is a common limitation for numerous studies mentioned in this review, it is necessary to tackle this boundary.

Mitochondrial delivery methods such as direct microinjection, cell-mediated delivery using tunneling nanotubes, vesicle- or liposome-mediated delivery and systemic delivery have been studied in various ways to improve mitochondrial absorption and transplantation efficiency. However, it has been reported that these delivery methods may interfere with transplantation efficacy [74]. A method of transplanting mitochondria by binding to a carrier protein was developed and succeeded in increasing the transplantation efficiency [84]. However, this method must be optimized according to the type of cell or tissue. In addition, specific delivery protocols and routes of administration must be developed and sufficient validation procedures are required. A large number of mitochondria can be transplanted at once by systemic injection, however, they can be implanted into unwanted tissues, and measuring this can cause other side effects.

Many groups have been pinpointed in this review, suggesting that targeting mitochondria is indeed a notable direction to head towards. However, the enigmatic mechanism orchestrating mitochondrial incorporation is yet to be fully uncovered. Although there are many challenges to be addressed, we foresee that mitochondrial transplantation will become a beneficial therapeutic choice for both physicians and surgeons to use in the treatment of diseases related to mitochondrial defects.

## Figures and Tables

**Table 1:** Research reports of mitochondrial internalization in neurological diseases.

Mitochondrial Source	Targeted Cell/Organs	Therapeutic Outcome	Delivery Method	Mitochondrial Concentration Range	Reference
Human hepatoma cells (HepG2 cells)	Human neuroblastoma cells (SH-SY5Y cells)	Increase in ATP contents, reduction of ROS production and apoptosis	Co-incubation	1.56~50 µg/mL different concentration range	[56]
Human hepatoma cells (HepG2 cells)	Neurotoxin-induced PD mouse brain	Rescue of mitochondrial function and decrease in cell death	Intravenous injection	0.5 mg/kg body weight	[56]
Rat pheochromo-cytoma cells (PC12 cells)	Rat pheochromo-cytoma cells (PC12 cells)	Reduction of ROS production and apoptotic cells	Co-incubation (peptide-mediated delivery)	105 µg/200 µL different concentration range	[57]
Rat pheochromo-cytoma cells (PC12 cells)	Neurotoxin- induced PD rat brain	Decrease in dopaminergic neuron loss	Direct injection	1.05 µg/each	[57]
Rat brain synaptosome	Human neuroblastoma cells (LAN5 cells)	Replacement of damaged mitochondria.	Co-incubation (synaptosome-based delivery)	2.5 × 10^7^~ 10.2 × 10^7^ particles/100 µL	[58]

Adenosine triphosphate (ATP); reactive oxygen species (ROS).
